# Compressed single-shot 3D photoacoustic imaging with a single-element transducer

**DOI:** 10.1016/j.pacs.2023.100570

**Published:** 2023-11-07

**Authors:** Bingbao Yan, Bowen Song, Gen Mu, Yubo Fan, Yanyu Zhao

**Affiliations:** Beijing Advanced Innovation Center for Biomedical Engineering, Key Laboratory for Biomechanics and Mechanobiology of Ministry of Education, School of Engineering Medicine, Beihang University, Beijing 100191, China

**Keywords:** Three-dimensional imaging, Single-shot imaging, Photoacoustic imaging, Compressive image reconstruction

## Abstract

Three-dimensional (3D) photoacoustic imaging (PAI) can provide rich information content and has gained increasingly more attention in various biomedical applications. However, current 3D PAI methods either involves pointwise scanning of the 3D volume using a single-element transducer, which can be time-consuming, or requires an array of transducers, which is known to be complex and expensive. By utilizing a 3D encoder and compressed sensing techniques, we develop a new imaging modality that is capable of single-shot 3D PAI using a single-element transducer. The proposed method is validated with phantom study, which demonstrates single-shot 3D imaging of different objects and 3D tracking of a moving object. After one-time calibration, while the system could perform single-shot 3D imaging for different objects, the calibration could remain effective over 7 days, which is highly beneficial for practical translation. Overall, the experimental results showcase the potential of this technique for both scientific research and clinical applications.

## 1 Introduction

Photoacoustic imaging (PAI) has gained increasingly more attention in biomedical applications. It can probe absorption contrast and generate three-dimensional (3D) images, providing rich information content of the object. PAI has shown great potential in various biomedical scenarios, such as small animal research [1], [2], [3], [4], [5], [6], [7], stain-free histopathology [8], breast cancer monitoring [9], and functional human brain monitoring [10].

**Fig. 1:**
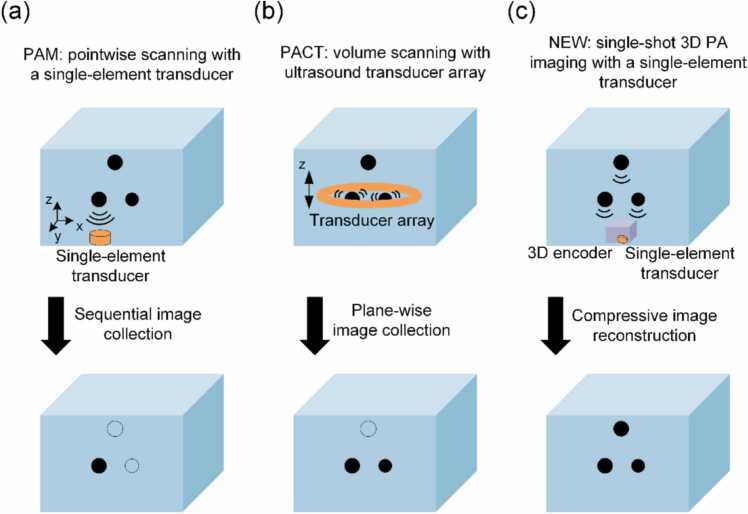
Three modalities of 3D photoacoustic imaging. (a) Conventional PA microscopy uses a single-element transducer, but requires pointwise scanning to obtain a 3D image. (b) PACT detects PA waves in parallel, but requires hundreds of transducer elements and data acquisition channels which are complex and expensive. (c) With a 3D encoder and compressive image reconstruction, the proposed method is capable of single-shot 3D photoacoustic imaging using a single-element transducer.

In order to generate a 3D image of the object, conventional PAI techniques typically require scanning and sequential data collection, which could be time-consuming or involves complex hardware. For example, as shown in Fig. 1(a), while photoacoustic microscopy (PAM) can perform 3D imaging via sequential data collection with a single-element transducer, it requires pointwise scanning of the entire volume, which is time-consuming [11], [12]. Different from PAM, photoacoustic computed tomography (PACT) usually conducts 3D imaging via volume scanning with an ultrasonic transducer array, as shown in Fig. 1(b). While the image collection of PACT is faster than that of PAM, the required detection hardware, which contains hundreds of transducer elements and data acquisition channels, is much more complex and expensive, severely limiting practical translation [1], [9], [10], [12], [13], [14].

To resolve the above dilemma between system complexity and imaging throughput in 3D PAI, compressive sensing has been introduced to reconstruct 3D images with under-sampled PA signals [15], [16], [17], [18], [19]. For instance, Provost and Lesage compared multiple sparse techniques for the reconstruction in PAI [15]. Guo et al. adapted compressive sensing for 3D PAI using wavelet basis and a linear transducer array [19]. Huynh et al. combined a single-pixel camera and a planar Fabry–Pérot ultrasound sensor for compressed 3D PAI with decreased number of measurements [17]. Particularly, for the first time, Lan et al. directly introduced a shape prior, developed an untrained neural network, and achieved compressed 3D PAI using a 128-element transducer array [16]. However, these methods either involves data collection with multiple measurements or requires complex transducer array. In addition, it’s worth noting that recently Li et al. and Zhao et al. achieved single-shot PAI with a single-element transducer, but the acquired measurements were limited in 2D [20], [21].

To address the above bottleneck, in this work, we take advantage of compressive sensing techniques and develop a new photoacoustic imaging method capable of single-shot 3D imaging with a single-element transducer. We refer to this method as SS3D-PAI (i.e., single-shot 3D photoacoustic imaging with single-element transducer). Compared to previous compressive photoacoustic imaging methods that can generate 3D images from multiple measurements with transducer array [15], [16], [17], [18], [19], the proposed method only requires a single laser shot and a single-element transducer. In addition, compared to recent works that are limited in 2D [20], [21], the proposed method can perform 3D imaging with a single laser shot and a single-element transducer, which substantially improves the imaging capacity.

## 2 Single-shot 3D photoacoustic imaging with a single-element transducer

As shown in Fig. 1(c), with the proposed method, PA signals from the objects in the entire volume are collected simultaneously by a single-element transducer via a 3D encoder. After the PA signals enter the encoder, the spatial information of the objects is transformed into unique temporal features and then collected by the single-element transducer. In other words, the 3D spatial information contained in those PA signals is first encoded into a unique time series (collected by the single-element transducer), and the 3D image can then be obtained by compressive image reconstruction.

To facilitate compressive image reconstruction, the 3D encoder should be able to project the spatial information of different 3D locations onto a set of incoherent basis functions. Inspired by previous works of Li et al. and Zhao et al., in this work the 3D encoder is composed of a right-angle prism and a cylindrical ultrasonic pipe (as shown in experimental setup below) [20], [21]. It is worth noting that our work is different from previous ones in three folds: 1) the ultrasonic pipe has a larger aperture which effectively collects a larger portion of PA signals, leading to higher signal-to-noise ratio (SNR); 2) the ultrasonic pipe is customized to have a circular aperture rather than hexagonal in Zhao et al., which sufficiently scrambles the PA waves inside the encoder and enables the use of a larger transducer size while maintaining spatial resolution much finer than the size of transducer (additionally, experimental data shows improved lateral resolution with cylindrical pipe than hexagonal pipe, details in Supplementary Note 1); 3) our work is able to conduct single-shot 3D imaging using a single-element transducer, while the previous works were limited in 2D.

**Fig. 2:**
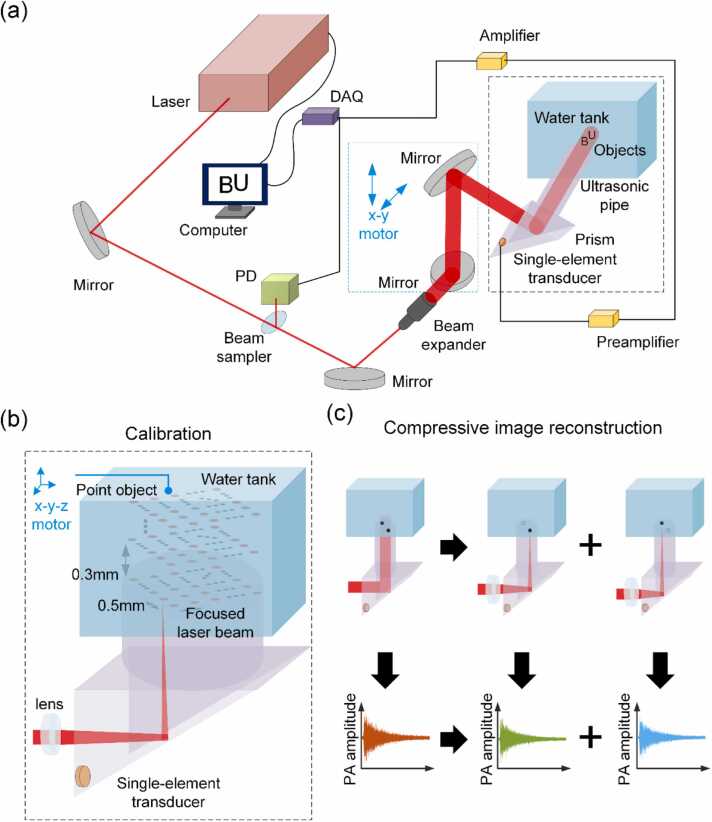
SS3D-PAI system and compressive image reconstruction. (a) Schematic of the experimental system. (b) Pointwise calibration of SS3D-PAI. (c) Compressive image reconstruction.

As shown in Fig. 2(a), an experimental system was built to demonstrate the proposed single-shot 3D PA imaging with a single-element transducer. The customized right-angle prism (9 cm edge length) and cylindrical ultrasonic pipe (3 cm aperture diameter, 30 cm length) were both made of UV-fused silica, which had negligible attenuation over the acoustic detection pathlength. A flat single-element transducer with central frequency of 1 MHz (−6 dB bandwidth of 84.66%) and element size of 1.3 cm diameter (V103-RM, Olympus IMS, Japan) was used as ultrasonic detector and placed at the corner of the prism. A thin layer of coupling gel (SWC-2, Olympus IMS, Japan) was added between the prism and the transducer to facilitate ultrasound transmission. Particularly, the SWC-2 coupling gel is different from regular gels used in clinical ultrasound and is visually like thick honey. We found a thin layer of it to be very helpful in our experiment: since it almost does not dry over time, one would not need to add the coupling gel and re-attach the transducer repeatedly between experiments. In addition, a nanosecond laser with 670 nm wavelength, 2 mJ pulse energy and 5 ns pulse width (10 Hz repetition rate, NT350, EKSPLA, Lithuania) was used as light source. The laser beam was partially reflected by a glass beam sampler onto a photodiode (PD) for the correction of fluctuations in pulse energy. For single-shot 3D imaging, the laser beam was expanded by a beam expander, and then reflected towards the imaging volume to provide widefield illumination. The generated PA signals were amplified by two sequential amplifiers (ZX60–43-S+ and ZFL-500LN-BNC+, respectively, Mini-Circuits, US) and then collected by a digital acquisition (DAQ) card (60 MHz bandwidth, M2p.5961-x4, Spectrum Instrumentation, Germany). For single-shot 3D imaging, the 3D image of the objects was reconstructed using the acquired single-shot data as well as a calibration dataset obtained in advance.

The calibration dataset was obtained through pointwise scanning of the imaging volume, as shown in Fig. 2(b). During calibration, the expanded beam was focused through a lens (500 mm focal length, 54 mm diameter, Grand Unified Optics, China) and illuminated onto a “point object” made of black rubber. The point object had a size of 0.5 mm diameter, which was much smaller than the acoustic wavelength (approximately 1.5 mm) at the central frequency of the transducer. In order to scan across the entire volume, both the focused laser beam and the calibration object were moved simultaneously in a synchronized manner by motorized scanners (PP110–75XYZ, PDV, China). In experiments, the calibration volume was 15 × 15 × 6 mm, and the scanning increments were 0.5 mm in the X-Y plane and 0.3 mm along the Z axis. At each scanning point, the data acquisition was repeated 2 times and averaged to increase SNR. The entire calibration process took 3 h (with 10 Hz laser repetition rate). It is worth noting that the calibration only needs to be conducted once, and can be applied to single-shot 3D imaging of different objects. In addition, as discussed in following section, once completed, the calibration can remain effective for at least 7 days.

The compressive imaging reconstruction for the proposed method is illustrated in Fig. 2(c). Since the recorded PA signal on the single-element transducer is composed of signals of different objects in the 3D volume, it can be decomposed back into a linear combination of those individual signals. The 3D image can therefore be obtained by resolving the inverse problem using a two-step iterative shrinkage/thresholding (TwIST) algorithm [22]. Specifically, the PA signal recorded on the transducer, denoted as y, can be represented as a linear combination of the 3D calibration basis, denoted as $k_{i}$, as shown in Eq. (1),

(1) $$y=\sum_{i=1}^{N}k_{i}x_{i}$$

where i, N, and $x_{i}$ denote calibrated pixel index, the total number of calibrated pixel locations, and the pixel intensity of the 3D image, respectively. Eq. (1) can be cast to a matrix form for simplicity: y=Kx, where $K=\left[k_{1},k_{2},\ldots ,k_{N}\right]$ and $x=\left[x_{1},x_{2},\ldots ,x_{N}\right]$. The 3D image x can be recovered by minimizing the objective function in Eq. (2), where $\Phi_{\mathrm{TV}}(x)$ is the total variation regularization term, and λ is the regularization parameter. The effect of tuning the regularization parameter on the reconstructed images is demonstrated in Supplementary Note 2.

(2) $$\hat{x}={\arg\underset{x}{\min}\frac{1}{2}\left\|\;y-\mathrm{Kx}\right\|}^{2}+\lambda \Phi_{\mathrm{TV}}(x)$$

In terms of time cost, in our experiments, the image reconstruction with TwIST algorithm took 88.9 ± 0.7 s (repeated 10 times) using a desktop computer with Intel Xeon Silver 4210 R CPU (2.40 GHz) and 64 GB RAM.

## 3 Experimental validations

**Fig. 3:**
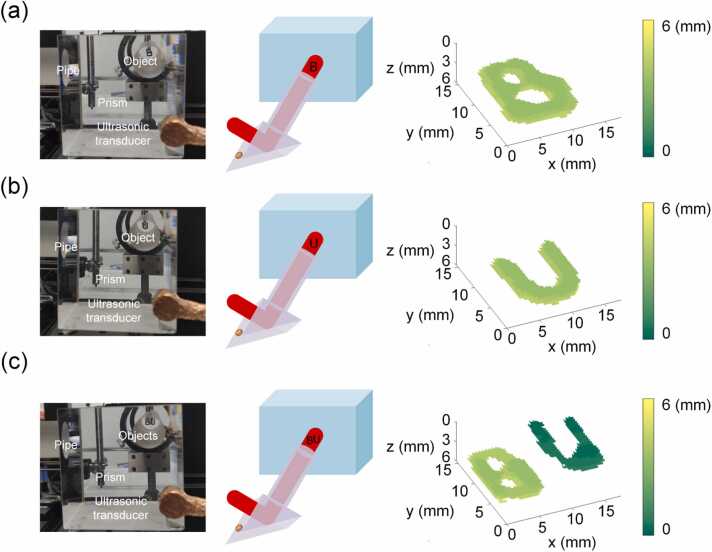
Validation of SS3D-PAI with different measurement objects. (a) Single-shot 3D imaging of a B-shaped object. (b) Single-shot 3D imaging of a U-shaped object. (c) Single-shot 3D imaging of two objects at different depths.

As proof-of-concept, the proposed method was first validated with a phantom study and then demonstrated for 3D tracking of a moving object. As shown in Fig. 3(a), a B-shaped black rubber object was placed inside the water tank and in front of the cylindrical ultrasonic pipe. The water tank had a 3 cm diameter hole where the pipe could fit in, and the gaps were sealed to prevent leakage. In addition, the single-element transducer, as shown in the picture, was placed at the corner of the right-angle prism, and enclosed by copper film for shielding. With a single-shot laser illumination and data collection by the single-element transducer, as shown in Fig. 3(a), the object was successfully reconstructed in 3D with both shape and depth information. Additionally, without repeated calibration, a different object (i.e., a U-shaped object made of black rubber) was also successfully reconstructed in 3D, as shown in Fig. 3(b). To further demonstrate the 3D imaging capability of the proposed method, two different objects (B-shaped and U-shaped, respectively) were placed at different depths and imaged in a single-shot using the system. In order to fit in the calibrated volume, the two objects were fabricated smaller than the ones used in earlier experiments in Fig. 3(a) and (b). As shown in Fig. 3(c), both objects as well as their depth information were successfully recovered with the proposed method.

**Fig. 4:**
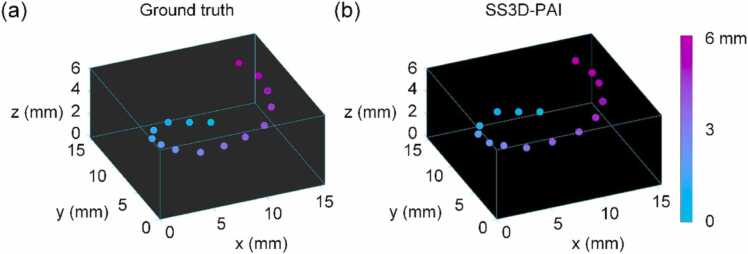
3D tracking of object with upward spiral motion. (a) Ground truth locations of the object. (b) Estimated locations with the proposed method.

In addition to single-shot 3D imaging of static objects, the proposed method can potentially be used to track moving objects in the calibrated volume. As proof-of-concept, the central point location of a single object with upward spiral motion was monitored with the proposed method. The object was a 5 mm black rubber disk with 1 mm thickness. Fig. 4 shows ground truth locations of the object at the time of imaging, as well as estimated locations obtained by the proposed method. The central positions rather than the reconstructed images were plotted since the object was relatively large compared to the imaging area. While the fluence of laser beam for the imaging was 0.64 mJ/cm^2^, the motion tracking can also be conducted for smaller objects using higher laser beam fluence (e.g., 5 mJ/cm^2^ for the widefield imaging in Zhang et al. [23]). The central position was estimated by respectively averaging the X, Y, and Z coordinates of the object pixels in the reconstructed image. The absolute position was determined relative to the origin of the calibrated 3D volume. The discrepancies between the estimated locations and the ground truth was −0.2 ± 0.4 mm, −0.6 ± 0.9 mm, and −0.1 ± 0.2 mm, for X, Y, and Z dimensions, respectively. The data shows that the proposed method was able to track the moving object with a single-shot laser pulse using a single-element transducer in the 3D volume.

**Fig. 5:**
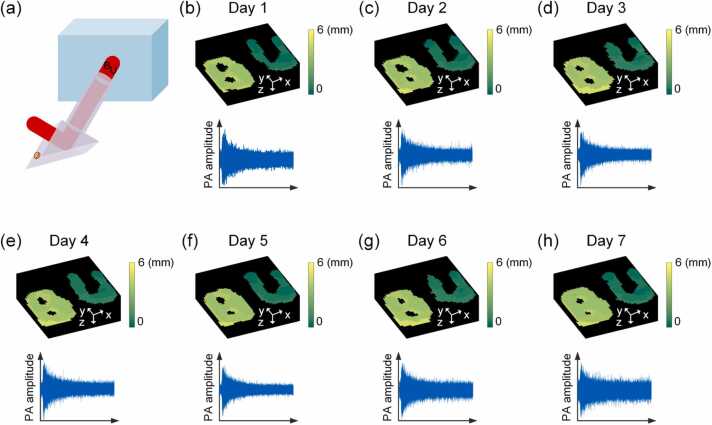
System calibration remains effective for 3D imaging over a time period of 7 days.

Hereinabove, it has been demonstrated that once calibrated, the system can be applied to 3D imaging of different objects as well as 3D tracking of moving object. For practical application, it would be ideal if the system calibration can remain effective for a long period of time. In order to get a sense of the time duration that the system calibration can stay effective, a 7-day experiment was conducted. Specifically, after a one-time calibration on Day 1, two different objects (B-shaped and U-shaped, respectively) placed at different depths were imaged using the proposed system, as shown in Fig. 5(a) and (b). With the same calibration obtained on Day 1, the objects were repeatedly measured every day in the following week, as shown in Fig. 5(c)-(h). The results demonstrate that once calibrated, the system calibration can remain effective for at least 7 days, which is highly beneficial for practical translation.

## 4 Discussion and conclusions

We have developed and validated SS3D-PAI as a new method capable of single-shot 3D photoacoustic imaging with a single-element transducer. The key to SS3D-PAI was the compressive sensing strategy which transformed 3D information of the object into unique temporal features of the collected PA signal. The transformation from spatial to temporal domain was enabled by a 3D encoder composed of a cylindrical ultrasonic pipe and a right-angle prism. The encoder was made of fused silica which had high temporal stability and negligible acoustic attenuation. After a one-time calibration, the system could perform single-shot 3D imaging for different objects, and the calibration could remain effective for at least 7 days. Such capability has not been demonstrated in previous literature, and may pave the way for practical application as well as translation. In addition, it’s important to note that the lateral resolution of the proposed method is fundamentally determined by the acoustic wavelength in the material of the encoder (e.g., fused silica) at the transducer’s central frequency [20], [21]. In contrast, since different objects on the axial direction is separated by different times of arrival, the axial resolution is determined by the wavelength in the medium (i.e., water) at the transducer's central frequency as well as bandwidth. As a result, the axial resolution should be finer than the lateral resolution with shorter wavelength in water than in fused silica. Additionally, with the 1 MHz transducer, the resolution of the imaging system was experimentally quantified to be approximately 2.5 mm for the lateral direction (close to half the acoustic wavelength in the encoder at the transducer's central frequency) and 0.8 mm for the axial direction (close to half the acoustic wavelength in water at the transducer's central frequency), respectively (details in Supplementary Note 3). Furthermore, while we currently use an iterative algorithm that took 88.9 s on average for each 3D image reconstruction, in the future, the reconstruction process can potentially be significantly accelerated by developing a deep learning model that does not involve iterative optimization and can directly output the reconstructed 3D image in a straightforward manner [24], [25], [26], [27], [28].

While the resolution of the proposed method is fundamentally determined by the acoustic wavelength at the transducer’s central frequency, it’s worth noting that the transducer size would also impact the image resolution. One can envision that if the transducer size is as big as the prism surface, the spatio-temporal encoding of the ultrasound signals would become in vain, leading to severely deteriorated resolution. On the other hand, if the transducer size is very small, it wouldn’t be able to collect as much signals, practically leading to low SNR and decreased resolution. While the optimal transducer size in practice is also related to its sensitivity as well as cost, in this work, with 90 mm edge length of the prism, a transducer of 13 mm diameter is used. We note that the transducer used in this work is off-the-shelf, low-cost, and commercially available, which is beneficial for potential clinical translations.

While the use of a larger pipe collects more signals and improves SNR, the critical angle between the pipe and water would impact the aperture over which the PA waves can effectively couple into the relay. In general, for a fixed point, more PA waves would couple into the fused silica ultrasonic pipe with an increasing pipe aperture, until the incidence angle (from the fixed point to the edge of the pipe) becomes greater than the critical angle. Quantitatively, the critical angle can be determined by the acoustic velocities of water and fused silica. The speed of sound is 5900 m/s in the fused silica pipe and 1500 m/s in water, respectively. The critical angle (denoted as $\theta_{c}$) satisfies $\sin\theta_{c}=\frac{v_{\mathrm{water}}}{v_{\mathrm{pipe}}}$, which leads to critical angle of 14.73°. In other words, PA waves beyond the critical angle would not be effectively collected into the pipe for SNR improvement. Moreover, note that the calibration (and imaging) was performed over a smaller aperture than the cylindrical pipe. In the compressive image reconstruction, since the recorded PA signal on the single-element transducer is composed of signals of different objects in the 3D volume, it is decomposed back into a linear combination of individual signals obtained by pointwise calibration to form an image. Given the single detector, as discussed in detail by Li et al. [20], the maximum number of resolvable pixels (i.e., the information capacity) is determined by signal SNR, time duration and temporal resolution, and would remain the same for different sizes of imaging area. In other words, with a larger imaging volume, the image reconstruction can still be conducted, but the information capacity would remain the same given the number of detectors is constant. In order to extract more information content in the imaging volume, one may need to increase the number of detectors as well. Additionally, since only PA waves within the critical angle can effectively couple into the pipe, the image reconstruction would become quite challenging if the imaging volume becomes significantly larger compared to the aperture.

SS3D-PAI enjoys several advantages over current PA methods. Compared to PA microscopy (PAM) techniques that also use a single-element transducer for 3D imaging, SS3D-PAI can perform single-shot 3D imaging, whereas PAM requires pointwise scanning over the entire 3D volume. While photoacoustic computed tomography (PACT) may conduct single-shot 3D imaging without pointwise scanning, it requires an ultrasonic transducer array which is known to be complex and expensive [1], [10]. In contrast, SS3D-PAI only requires a single-element transducer to achieve single-shot 3D imaging. Furthermore, compared to other works that also used compressive sensing, the proposed method is capable of single-shot 3D imaging with a single-element transducer, while previous methods either required an array of ultrasound transducers, or were limited in 2D [16], [20], [21]. Additionally, it is worth noting that during the submission process of this work, we became aware of a preprint that also demonstrates 3D photoacoustic imaging using a spatio-temporal encoder [23]. We note that the preprint and this work are different in several key aspects. In the preprint work, the imaging volume is 8 × 8 × 3.6 mm, with 18 mm diameter of the cylindrical pipe. In contrast, this work has a larger imaging volume (i.e., 15 ×15 ×6 mm, approximately 6 × larger), and a larger diameter of the cylindrical pipe (i.e., 30 mm). The larger diameter of the cylindrical pipe allows collection of more PA signals from the objects into the pipe, which would help improve SNR compared to a smaller aperture. Moreover, the active aperture size of the transducer is 0.4 × 0.4 mm in the preprint work and 13 mm in diameter in this work. The larger transducer aperture also allows collection of more ultrasonic signals from the prism, which is beneficial for the SNR. Regarding resolution, the preprint had lateral resolution of 0.56 mm and axial resolution of 0.13 mm with a 40 MHz transducer. In contrast, this work achieved lateral resolution of 2.5 mm and axial resolution of 0.8 mm with a 1 MHz transducer.

This work has a number of important implications for scientific research and clinical applications. For example, 3D brain imaging is of fundamental significance for neuroscience as well as cognitive sciences [12], [29], [30], [31]. Current methods such as confocal microscopy and two-photon microscopy are limited to small imaging volume and require exogenous contrast agents. In contrast, the proposed method can potentially provide both large imaging volume and label-free functional imaging. In addition, given sufficient laser pulse repetition rate, the proposed method can potentially achieve kilohertz 3D photoacoustic imaging using a single-element transducer, which may benefit studies focusing on ultrafast biological phenomena, such as large-scale cerebral cortex activity, cardiac motion, and freely behaving organisms [32]. Therefore, in the future, SS3D-PAI may be a powerful tool for scientific research. Regarding clinical applications, the proposed method may also enable preoperative deep vascular imaging for noninvasive diagnosis and treatment of clinical blood vessels, which would significantly reduce system cost and complexity than current PACT-based modalities [33], [34].

In summary, we have developed SS3D-PAI, a new imaging modality that is capable of single-shot 3D photoacoustic imaging using a single-element transducer without the need of transducer array or pointwise scanning of the imaging objects, which successfully overcomes the major limitations of previous methods. In the future, SS3D-PAI can be potentially useful for both scientific research and clinical applications.

## Declaration of Competing Interest

The authors declare that they have no known competing financial interests or personal relationships that could have appeared to influence the work reported in this paper.

## Data Availability

Data will be made available on request.
