# Supplementary material for: Compressed single-shot 3D photoacoustic imaging with a single-element transducer
Source: Photoacoustics. 2023 Nov 7;34:100570. doi: 10.1016/j.pacs.2023.100570 (PMC10661598; doi:10.1016/j.pacs.2023.100570)
Supplement: Supplementary file 1 — Supplementary material [file mmc1.docx]

*Supplementary Information*

**Compressed single-shot 3D photoacoustic imaging with a single-element transducer**

Bingbao Yan^1^, Bowen Song^1^, Gen Mu^1^, Yubo Fan^1,^*, Yanyu Zhao^1,^*

^1^Beijing Advanced Innovation Center for Biomedical Engineering, Key Laboratory for Biomechanics and Mechanobiology of Ministry of Education, School of Engineering Medicine, Beihang University, Beijing 100191, China.

*** Corresponding authors**.

**E-mail address:** [yubofan@buaa.edu.cn](mailto:yubofan@buaa.edu.cn) (Y. Fan), [yanyuzhao@buaa.edu.cn](mailto:yanyuzhao@buaa.edu.cn) (Y. Zhao)

**Supplementary Note 1 – Shape choice of the ultrasonic pipe**

We chose cylindrical shape for the ultrasonic pipe under the rational that the cylindrical shape would help sufficiently scramble the PA waves inside the pipe and enable better resolution in practice. Specifically, in addition to the 3D encoder demonstrated using cylindrical pipe, we also made its hexagonal counterpart (with 3 cm hexagonal aperture). We then experimentally quantified the imaging system resolution respectively using the hexagonal and the cylindrical pipe. With the hexagonal pipe, for the lateral resolution, image reconstruction was conducted for two points with different distances on the X-Y plane. The reconstructed images and their line profiles are shown in Fig. S1(a) below. The data shows that the imaging system with hexagonal pipe has a lateral resolution of approximately 3.2 mm, which is much larger than half the acoustic wavelength in fused silica at the transducer's central frequency. For the axial resolution, image reconstruction was conducted for two points with different distances on the X-Z plane. The reconstructed images and their line profiles are shown in Fig. S1(b) below. The data shows that the imaging system has an axial resolution of approximately 0.8 mm, which is close to half the acoustic wavelength in water at the transducer's central frequency. With the cylindrical pipe, we also experimentally quantified the lateral and axial resolution (details in Supplementary Note 3). Briefly, the experimental data shows that the imaging system with cylindrical pipe has a lateral resolution of approximately 2.5 mm, which is close to half the acoustic wavelength in the encoder at the transducer's central frequency. Additionally, the imaging system with cylindrical pipe also has an axial resolution of approximately 0.8 mm, which is close to half the acoustic wavelength in water at the transducer's central frequency. Overall, the experimental data shows that the imaging system has nearly the same axial resolution with both hexagonal and cylindrical pipes. This is what one would expect, because the ability to separate different objects on the axial direction comes from signal time of arrival, which is determined by the acoustic wavelength of the medium at the transducer's central frequency. On the other hand, the experimental data also shows that the imaging system has better lateral resolution with the cylindrical pipe compared to that with the hexagonal pipe, suggesting the shape choice of cylindrical over hexagonal.


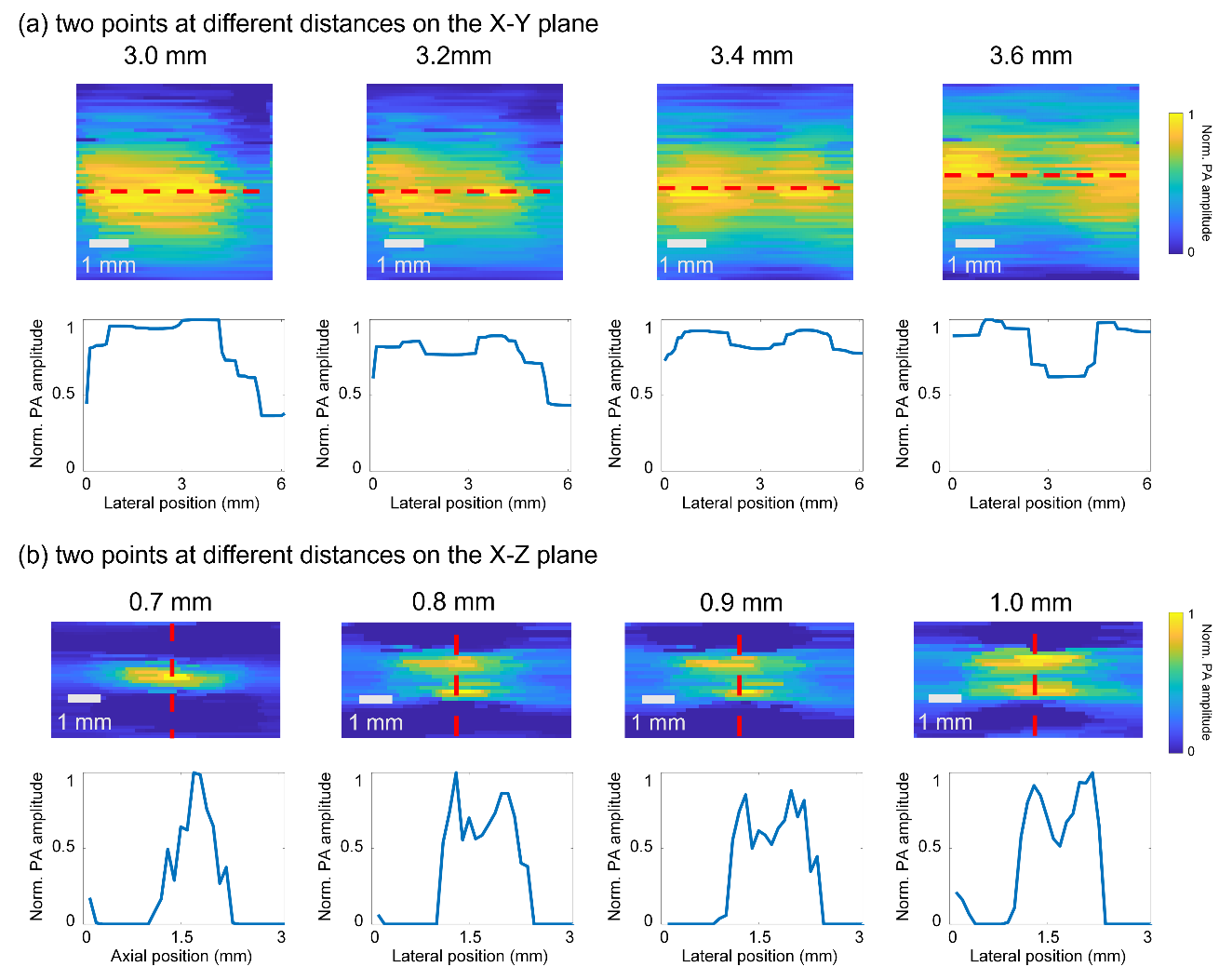


Fig. S1 (a) Two points with different distances on the X-Y plane and their line profiles for lateral resolution. (b) Two points with different distances on the X-Z plane and their line profiles for axial resolution.

**Supplementary Note 2 – Effect of tuning the regularization parameter on the reconstructed images**

In our study, we used the regularization parameter of λ=1e-4 for the TwIST algorithm. That being said, in the image reconstruction, we found that even in a wide range of [1e-10, 1e10], the choice of regularization parameter did not have major impact on the reconstructed images. As shown in Fig. S2 below, the reconstructed images appear quite similar with different regularization parameters from 1e-10 to 1e10.


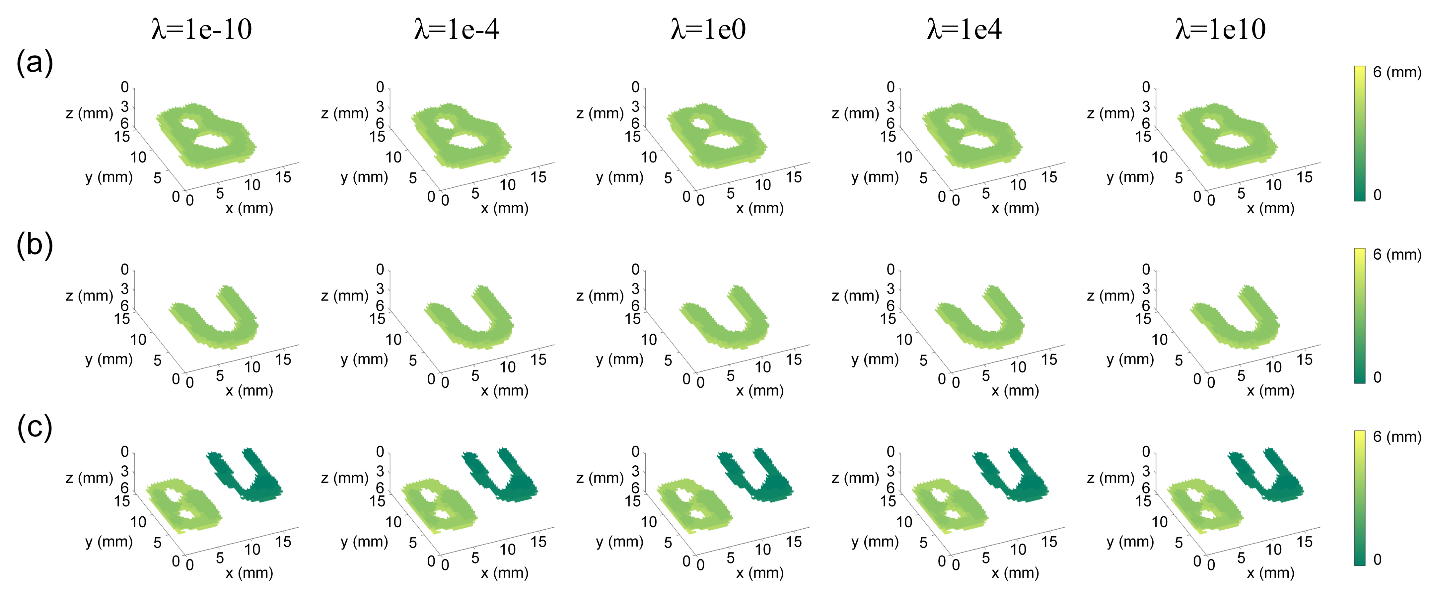


Fig. S2 Reconstructed images with different values of the regularization parameter.

**Supplementary Note 3 – Experimental quantification of lateral and axial resolution of the imaging system**

We experimentally quantified the lateral and axial resolution of our system. Specifically, For the lateral resolution, image reconstruction was conducted for two points with different distances on the X-Y plane. The reconstructed images and their line profiles are shown in Fig. S3(a). The data shows that the imaging system has a lateral resolution of approximately 2.5 mm, which is close to half the acoustic wavelength in the encoder at the transducer's central frequency. Additionally, for the axial resolution, image reconstruction was conducted for two points with different distances on the X-Z plane. The reconstructed images and their line profiles are shown in Fig. S3(b). The data shows that the imaging system has an axial resolution of approximately 0.8 mm, which is close to half the acoustic wavelength in water at the transducer's central frequency.


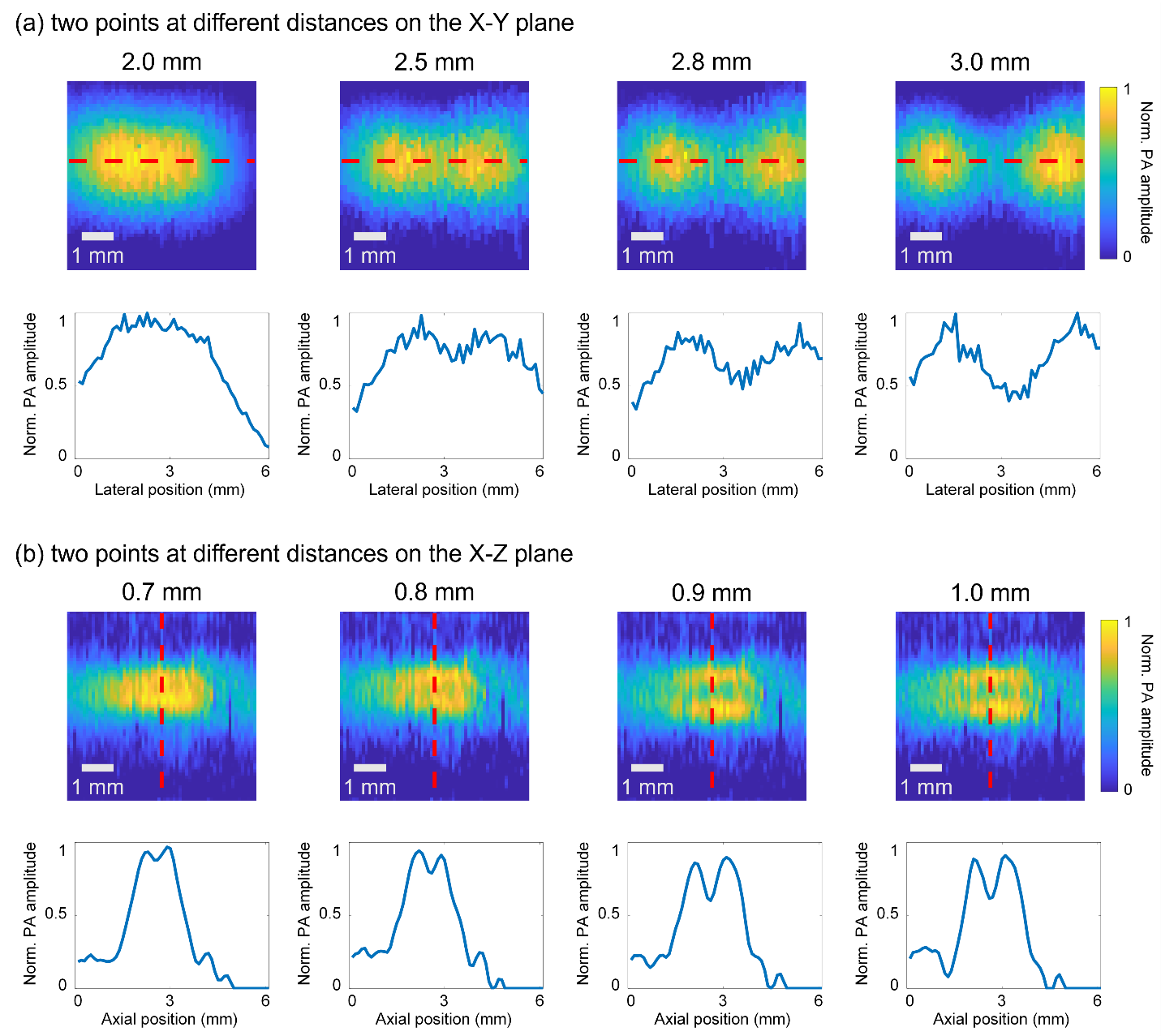


Fig. S3 (a) Two points with different distances on the X-Y plane and their line profiles for lateral resolution. (b) Two points with different distances on the X-Z plane and their line profiles for axial resolution.
